# Metabolic power and energy expenditure in the German Bundesliga

**DOI:** 10.3389/fphys.2023.1142324

**Published:** 2023-03-22

**Authors:** Jan Venzke, Hendrik Weber, Marc Schlipsing, Jan Salmen, Petra Platen

**Affiliations:** ^1^ Department of Sports Medicine and Sports Nutrition, Ruhr University Bochum, Bochum, Germany; ^2^ Sportec Solutions GmbH (a DFL Company), Frankfurt, Germany; ^3^ Athlens GmbH, Bochum, Germany

**Keywords:** activity profile, team sports, energetics, video match analysis, acceleration, intermittent activity, external load

## Abstract

The aims of the study were to analyze metabolic power (MP) and MP derived parameters for different positions in the German Soccer Bundesliga and to evaluate if classification of high-intensity is more suited using the metabolic power approach instead of using traditional speed-based methods. 1,345 video match analysis (25 Hz) datasets from 380 players in 96 matches of the German first Bundesliga were gathered by an automatic player tracking system. Displacement (speed, acceleration, distance) and energetic (MP, energy expenditure) variables were determined. Intensity was classified utilizing conventional thresholds. Metabolic and running profiles were compared among six positional groups and between the halves of the match respectively (one-way ANOVA). Further, time spent, distance covered and energy expended at high speed (>15.5 km h^−1^) and high acceleration (>2 m s^−2^) were compared to those at high MP (>20 W kg^−1^) (one-way ANOVA) for evaluating if metabolic power is more suited to describe intensity in team sports. Main findings are that central-attacking midfielders (CAM) and central midfielders (CM) expended more energy (CAM: 59.8 ± 4.2 kJ kg^−1^; CM: 59.6 ± 3.6 kJ kg^−1^) and covered more distance (CAM: 11,494 ± 765 m; CM: 11,445 ± 638 m) than all other positions (*p* < 0.001). In the whole group of players, less time (t) and less energy were expended (EE) in high speed (t: 302 ± 84 s; EE: 10.1 ± 2.9 kJ kg^−1^) and at high acceleration zones (t: 147 ± 24 s; EE: 5.5 ± 1.2 kJ kg^−1^) when compared to high MP zone (t: 617 ± 141 s; EE: 20.0 ± 4.4 kJ kg^−1^) (*p* < 0.001). Furthermore, players covered more distance at high MP (2,464 ± 597 m) than at high speed (1,638 ± 458 m) and much less at high acceleration (359 ± 67 m) (*p* < 0.001). The higher activity profiles of CAM and CM compared to the other positions indicate the need for higher developed physiological performance in players of these positions. High intensity activities should be interpreted differently when using MP and displacement parameters as indicators of high intensity in soccer.

## Introduction

Soccer is characterized by its high-intensity and intermittent nature in which players are required to perform several short bursts of varying high-intensity actions which place a high level of stress on the body. Time-motion analysis in soccer is widely used to determine kinematic variables like distance, average and maximal speed and movement across different speed zones (Bourdon et al., 2017; Miguel et al., 2021). Supposedly, the overall work in field sports can be expressed as total distance covered, based on the flawed assumption that this measure represents the energy expenditure regardless of movement speed (Carling et al., 2008), while the movement speed itself is assumed and used to signify exercise intensity (Bangsbo et al., 1991). However, these parameters do not consider fluctuations in movement speed, which contribute directly to energy cost (Polglaze et al., 2018). Especially, in highly erratic exercise episodes where only submaximal speeds are reached (Akenhead et al., 2014) the conventional speed approach lacks validity. To accelerate is more energetically demanding than to maintain speed (Varley and Aughey, 2013) but at different starting speeds the metabolic work differs (Osgnach et al., 2010). Therefore, estimations of intensity in team sports like soccer must incorporate acceleration as well as speed.

Consequently, a theoretical model estimating the energy cost of accelerated, decelerated and constant running, which are common movement patterns in team sports has been proposed. This model considers accelerated running on a flat surface as energetically equivalent to running with a constant speed on an incline where the angle of the incline is similar to the forward lean of the human body while accelerating (Di Prampero et al., 2005). Hence, speed and acceleration can be used to estimate the instantaneous energy requirement and O_2_ demand in time series data (Di Prampero and Osgnach, 2018). Thresholds can be based on the assessment of physiological variables (e.g., V̇O_2_max, anaerobic threshold) to calculate time, distance and energy spent in high- and low-intensity zones. Thus, the metabolic power approach is probably a more accurate approach than the speed and/or acceleration approach alone (Di Prampero and Osgnach, 2018). Recent studies conducted metabolic power analyses in different sports like Rugby (Kempton et al., 2015), Australian Football (Coutts et al., 2015), Hockey (Polglaze et al., 2018) and Soccer (Osgnach et al., 2010) and highlighted the advantages: It was, for instance, shown that high-speed running distance may underestimate the high-intensity demands of match-play when compared to high-metabolic power distance (Coutts et al., 2015; Kempton et al., 2015; Polglaze et al., 2018). Until now, only the early study from Osgnach et al. (2010) analyzed MP in a top-level international soccer league, namely the Italian Serie A. No further top-level leagues, and especially no more recent seasons, were analyzed since then, although soccer match intensity seems to have increased over the last 10 or more years (Radziminski and Jastrzebski, 2021).

Using speed and acceleration zone analysis, several studies in soccer reported differences between playing positions in total distance covered and distance covered in the various speed and acceleration zones (Bradley et al., 2010; Di Salvo et al., 2013; Varley and Aughey, 2013; Mallo et al., 2015). Generally, wide midfielders (11,496 m) and central midfielders (11,487 m) covered more total distance in official matches than wide defenders (10,639 m), central defenders (9,901 m) and forwards (10,451 m) (Di Salvo et al., 2013). Central defenders covered the least distance in high-speed running (>19.8 km/h) (343 m) (Mallo et al., 2015) while wide midfielders showed the most high-speed running (533 m) and sprinting distance (382 m) (Di Salvo et al., 2013). Analyzing all positions together, more distance was covered in the first half of the game compared to the second half (Metaxas, 2021) assuming the occurrence of fatigue. All of these studies used the traditional distance/high-speed running approach to quantify volume and intensity, however, using this approach neglects one common aspect of team sports—the perpetual changes in speed. Traditional time-motion analyses generally report on acceleration characteristics separately from speed, however, the energetic demands in team sports are determined by the interaction between these two parameters (di Prampero et al., 2005; Polglaze and Hoppe, 2019). Furthermore, accelerations and decelerations are treated equally in most cases, even though deceleration is less demanding than acceleration. The interplay between speed and acceleration, as well as the magnitude, duration, and direction (positive or negative) of accelerations, are all considered in the calculation of metabolic power (Polglaze and Hoppe, 2019).

Consequently, the aims of this study were to analyze more recent metabolic power estimates in European top-league soccer players of different positions and to compare traditional speed- and acceleration-based approaches for identifying high-intensity activity with the metabolic power approach for the same purpose. It is hypothesized that 1) positional differences in volume and intensity parameters exist in volume and intensity parameters, and that 2) classification of high-intensity is more suited using the metabolic power approach.

## Materials and methods

### Data analysis

All matches from three teams of the German Bundesliga (first division) across the 2016/17 season plus their respective opponent teams were analyzed. In total, 1,345 discrete player data sets from 96 matches were gathered from 380 players (25.3 ± 4.0 years, 183.0 ± 6.3 cm, 77.7 ± 6.6 kg). Written informed consent was obtained prior to participation. The study was approved by the Ethics Commission of the Faculty of Sport Science of the Ruhr University Bochum. Data was collected by a semi-automatic camera match analysis system. Two cameras (25 Hz), one for each half of the pitch, are permanently installed in all stadiums of the league. The system calculates two-dimensional position data (x and y). High reliability (ICC ≥ 0.98) in reanalyzing metabolic power data using this system has been demonstrated previously (Beato and Jamil, 2018). Good validity over a range of soccer specific movements, speeds and sprints have been demonstrated (Redwoord-Brown et al., 2012). This system showed similar accuracy in measuring the athlete’s position (56 ± 16 cm) compared to Global Positioning System (96 ± 49 cm) and Local Positioning System (23 ± 7 cm) (Linke et al., 2018).

All datasets were grouped into six player positions—wing-back (WB; 68 players, 230 matches), center-back (CB; 82 players, 394 matches), wide midfielder (WM; 88 players, 226 matches), central-midfielder (CM; 79 players, 291 matches), striker (ST; 42 players, 158 matches) and central-attacking-midfielder (CAM; 21 players, 46 matches). Goalkeepers and player who did not play the entire match were not included in this analysis.

### Match activities

Speed, acceleration and metabolic power (MP) variables were determined and categorized using established thresholds (Osgnach et al., 2010). Also, distance covered, time spent (TS) and energy expended in each speed, acceleration and metabolic power category were identified. Metabolic power and energy cost were calculated using previously outlined equations (Di Prampero et al., 2005). Total distance (TD), energy expenditure (EE), average speed and average MP were determined for the net- and total-playing time for each position. The net playing time was defined as the period of active play, ergo when the ball was on the pitch and there was no interruption due to foul play or freekicks. Additionally, equivalent distance (ED), representing the distance the athlete would have run at a steady pace using the total energy spent over the match (Osgnach et al., 2010) and the equivalent distance index (EDI), which stands for the ratio between ED and TD, were determined. A higher EDI indicates a more intermittent activity of higher intensity (Polglaze et al., 2018). The anaerobic index (AI), representing the ratio between the energy expenditure above a certain threshold and the total energy expenditure throughout the whole match, was also calculated. The respective threshold generally used is either the anaerobic threshold or a certain percent of the V̇O_2_max. As the individual fitness status of the players was not available, this threshold was set at a constant 20 W kg^−1^ for all players, corresponding to a V̇O_2_ of approximately 57 ml kg^−1^ min^−1^ (Osgnach et al., 2010). According to Osgnach et al. (2010), the constant (KT) for running on a grassy terrain was set at 1.29 (Osgnach et al., 2010).

In the di Prampero model constant speed running at 15.5 km h^−1^ represents a power output of 20.0 W kg^−1^ which is similar to an acceleration of 2 m s^−2^ when the starting speed is 5.4 km h^−1^. Therefore, thresholds for high speed, high acceleration and high MP were set at v > 15.5 km h^−1^, a > 2 m s^−2^ and MP > 20.0 W kg^−1^, respectively (Polglaze et al., 2018). EDI, EE, average MP and EE over the defined metabolic power threshold (>20 W kg^−1^) were chosen to evaluate differences and similarities between positional groups. Mean MP, EE, distance covered and high-power energy expenditure were calculated for each half and compared between halves. Furthermore, total distance (TD), EE, average speed, average MP, ED, EDI and AI were compared between positions. Time spent (TS), TD and EE over defined high-intensity threshold were evaluated and compared between player positions. Based on these parameters, we evaluated the suitability for estimating high-intensity demands in each approach (speed, acceleration, metabolic power).

### Statistical analysis

Data is presented as mean ± standard deviation and was calculated using SPSS 25.0 (IBM, Chicago, IL, United States). Assumptions of normality were verified prior to parametric statistical analysis using the Kolmogorov-Smirnov Test. Levene’s test was used to assess the equality of variances. To compare player positions and team placements a one-way ANOVA and Bonferroni’s post-hoc test to identify differences were used. To evaluate the magnitude, partial η^2^ was used. The ranges for the size of the effect were set at 0.01 (small effect), 0.06 (medium effect) and 0.14 (large effect) (Cohen, 1988). Paired t-tests were used to compare match running performance and energetic characteristics between halves, Cohen’s d was used to describe effect size. The ranges for the size of the effect were set at: <0.2 (trivial effect), 0.2–0.6 (small effect), 0.6–0.1.2 (moderate effect); 1.2–2.0 (large effect) and > 2.0 (very large effect) as proposed for sports science (Hopkins, 2002). Statistical significance was set at *p* < 0.05.

## Results

### Positional differences

Mean playing time of all matches was 93:55 ± 2:13 min:s while net playing time reached 58:28 ± 4:12 min:s. Table 1 shows the analyzed data for each position and the entire team. In short, CM and CAM had the highest values in the parameters total distance, total energy expenditure, average speed, average metabolic power and equivalent distance, and CB showed the lowest values for each of the mentioned parameters. WM had lower values than CB and CAM but they covered more distance and expended more energy than WB and ST who presented similar values. The highest EDI was recorded for WM, followed by WB followed and ST. Further, CM, CAM and CB had a lower EDI than WM, WB and ST but did not differ among each other. The lowest value was shown for CB in the anaerobic index compared to the other positions while WM was the group with the greatest value, followed by ST who had a similar AI like CAM but higher values than WB, CM and CB, respectively. The comparison of the respective values of the net playing time revealed similar results in TD, EE, ED, EDI and AI.

**TABLE 1:** Displacement and energetic data for each position and the whole team (WB, widebacks; CB, centre-backs; WM, wide-midfielder; CM, central midfielder; CAM, central-attacking midfielder; ST, striker, n, number of discrete player data sets).

		All players (n = 1,345)			WB (*n* = 230)			CB (*n* = 394)			WM (*n* = 226)			CM (*n* = 291)			CAM (*n* = 46)			ST (*n* = 158)			Main effect	η^2^	Post-hoc tests
Total distance	m	10,531	±	950	10,389	±	566	9,755	±	615	10,807	±	772	11,445	±	638	11,494	±	765	10,319	±	1,018	*p* < 0.001	0.45	(CAM = CM) > WM > (WB = ST) > CB
Net total distance	m	8,111	±	929	7,976	±	593	7,427	±	640	8,237	±	766	9,017	±	702	8,984	±	866	7,915	±	938	*p* < 0.001	0.41	(CM = CAM) > WM > (WB = ST) > CB
Energy expenditure	kJ kg^−1^	56.79	±	5.28	55.27	±	3.18	51.65	±	3.59	57.33	±	4.29	59.61	±	3.61	59.81	±	4.21	53.98	±	5.37	*p* < 0.001	0.45	(CAM = CM) > WM > (WB = ST) > CB
Energy expenditure	kcal	1,047	±	98	1,012	±	89	1,037	±	95	1,035	±	102	1,093	±	79	1,093	±	110	1,039	±	107	*p* < 0.001	0.08	(CM = CAM) > (ST = CB = WM = WB)
Net energy expenditure	kJ kg^−1^	45.12	±	5.10	44.78	±	3.28	41.02	±	3.65	46.49	±	4.16	49.72	±	3.89	49.45	±	4.67	44.18	±	4.92	*p* < 0.001	0.41	(CM = CAM)>WM>(WB = ST)>CB
Average speed	m/s	1.87	±	0.17	1.87	±	0.12	1.82	±	0.16	1.93	±	0.13	2.03	±	0.12	2.04	±	0.15	1.91	±	0.17	*p* < 0.001	0.46	(CAM = CM) > (WM = ST = WB) > CB
Average metabolic power	W kg^−1^	10.08	±	0.93	10.00	±	0.57	9.27	±	0.63	10.44	±	0.75	10.90	±	0.62	10.93	±	0.81	9.92	±	0.94	*p* < 0.001	0.45	(CAM = CM) > (WM = WB = ST) > CB
Equivalent distance	m	12,228	±	1,132	12,129	±	687	11,252	±	772	12,673	±	924	13,223	±	778	13,271	±	907	12,037	±	1,156	*p* < 0.001	0.45	(CAM = CM) > WM > (WB = ST) > CB
Net equivalent distance	m	9,716	±	1,099	9,645	±	708	8,833	±	786	10,011	±	895	10,705	±	839	10,649	±	1,006	9,513	±	1,060	*p* < 0.001	0.41	(CM = CAM) > WM > (WB = ST) > CB
Equivalent distance index		1.16	±	0.02	1.17	±	0.02	1.16	±	0.02	1.17	±	0.02	1.16	±	0.02	1.16	±	0.01	1.17	±	0.02	*p* < 0.001	0.17	WM > (WB = ST) > (CM = CAM = CB)
Net equivalent distance index		1.20	±	0.03	1.21	±	0.02	1.09	±	0.02	1.22	±	0.03	1.19	±	0.02	1.19	±	0.01	1.20	±	0.03	*p* < 0.001	0.21	WM>(WB = ST)>(CM = CAM = CB)
Anaerobic index		0.35	±	0.05	0.35	±	0.05	0.32	±	0.04	0.37	±	0.04	0.37	±	0.06	0.36	±	0.05	0.35	±	0.06	*p* < 0.001	0.17	(WM = CM = CAM = WB = ST) > CB
Net anaerobic index		0.44	±	0.07	0.45	±	0.06	0.41	±	0.06	0.47	±	0.06	0.46	±	0.06	0.45	±	0.06	0.44	±	0.08	*p* < 0.001	0.14	(WM = CM = CAM = WB = ST) > CB

The time spent, distance covered and energy expended above the respective high-speed, -acceleration and -metabolic power thresholds for each position are outlined in Table 2. In short, WM, CAM and CM spent more time and covered more distance than the other positions above the high-speed threshold with a higher value for WM in distance covered in comparison to CM. ST and WB also spent more time and covered more distance than CB. A similarity of energy expenditure above the high-speed threshold was given in WM, CM and CAM who expended more energy than ST and WB. Furthermore, CB showed the smallest value. WM had the highest value of TS, TD and EE above the high acceleration threshold, while the lowest value was found for CB. All other positions had similar values. CAM and CM spent more time and covered more distance than the other positions above the metabolic power threshold, followed by WM, WB, ST and CB in this particular order. Further, ST and WB did not differ in distance covered above the MP threshold. Additionally, CB expended the least amount of energy above the MP threshold, while CM, CAM and WM did not differ among each other but expended more energy than WB and ST.

**TABLE 2:** Time spent, distance covered and energy extended in high-Intensity movement classified in high speed, high acceleration and high metabolic power for each position (WB, widebacks; CB, centre-backs; WM, wide-midfielder; CM, central midfielder; CAM, central-attacking midfielder; ST, striker).

		All players (*n* = 1,345)			WB (*n* = 230)			CB (*n* = 394)			WM (*n* = 226)			CM (*n* = 291)			CAM (*n* = 46)			ST (*n* = 158)			Main effect	η^2^	Post-hoc tests
High Speed (>15.5 km/h)																									
Time spent	min:s	5:02	±	1:24	5:07	±	0:58	3:40	±	0:51	6:00	±	0:59	5:50	±	1:14	6:04	±	1:16	5:13	±	1:09	*p* < 0.001	0.37	(CAM = WM = CM) > (ST = WB) > CB
Distance covered	M	1,638	±	458	1,687	±	332	1,189	±	279	1981	±	327	1845	±	402	1949	±	408	1728	±	380	*p* < 0.001	0.37	(WM = CAM = CM) > (ST = WB) > CB
Energy expended	kJ kg^−1^	10.05	±	2.87	10.35	±	2.28	7.54	±	1.85	12.10	±	2.24	11.09	±	2.62	11.72	±	2.68	10.53	±	2.73	*p* < 0.001	0.36	(WM = CAM = CM) > (ST = WB) > CB
High Acceleration (>2.0 m/s^2^)																									
Time spent	min:s	2:27	±	0:24	2:28	±	0:22	2:18	±	0:23	2:38	±	0:24	2:30	±	0:23	2:27	±	0:19	2:26	±	0:25	*p* < 0.001	0.05	WM>(CM = WB = CAM = ST)>CB
Distance covered	M	359	±	67	365	±	59	325	±	60	406	±	62	349	±	59	355	±	47	382	±	72	*p* < 0.001	0.12	WM > (ST = WB = CAM = CM) > CB
Energy expended	kJ kg^−1^	5.45	±	1.18	5.56	±	1.06	4.98	±	1.07	6.18	±	1.16	5.31	±	1.08	5.40	±	0.94	5.69	±	1.38	*p* < 0.001	0.12	WM > (ST = WB = CAM = CM) > CB
High metabolic power (>20.0 W/kg)																									
Time spent	min:s	10:17	±	2:21	10:09	±	1.36	8:38	±	1:32	10:57	±	1:51	12:20	±	2:17	11:53	±	2:21	9:32	±	2:13	*p* < 0.001	0.35	(CM = CAM) > WM > WB > ST > CB
Distance covered	M	2,464	±	597	2,456	±	424	1988	±	382	2,714	±	465	2,918	±	566	2,883	±	599	2,348	±	563	*p* < 0.001	0.36	(CM = CAM) > WM > (WB = ST) > CB
Energy expended	kJ kg^−1^	19.96	±	4.35	19.98	±	3.22	16.64	±	3.02	21.95	±	3.52	22.80	±	4.14	22.57	±	4.22	19.35	±	4.29	*p* < 0.001	0.31	(CM = CAM = WM) > (WB = ST) > CB
High speed vs. high metabolic power vs. high acceleration—main effect for each position																									
Time spent		MP > Speed > Acc			MP > Speed > Acc			MP > Speed > Acc			MP > Speed > Acc			MP > Speed > Acc			MP > Speed > Acc			MP > Speed > Acc			*p* < 0.001	0.93	
Distance covered		MP > Speed > Acc			MP > Speed > Acc			MP > Speed > Acc			MP > Speed > Acc			MP > Speed > Acc			MP > Speed > Acc			MP > Speed > Acc			*p* < 0.001	0.92	
Energy expended		MP > Speed > Acc			MP > Speed > Acc			MP > Speed > Acc			MP > Speed > Acc			MP > Speed > Acc			MP > Speed > Acc			MP > Speed > Acc			*p* < 0.001	0.94	

### Classification of high intensity

For each position, the time spent, distance covered and energy expended at high intensities were higher in the classification based on metabolic power compared to the speed classification (*p* < 0.05, η^2^ =0 .92–0.94, large effect).

### Differences of halftimes

**FIGURE 1:**
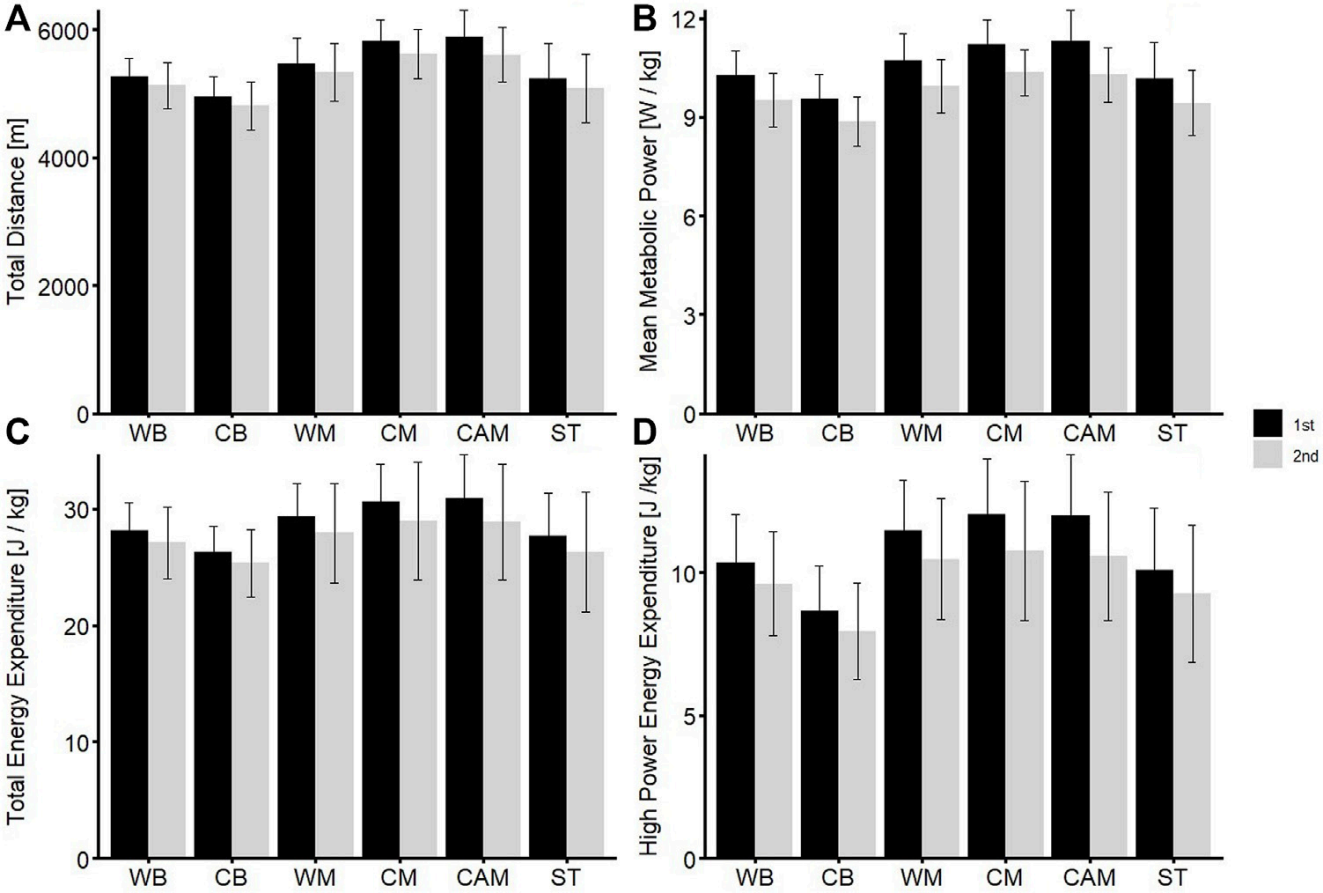
Comparison of volume and intensity parameters between the first and second halves of soccer matches for each position. The figure shows a significant decrease in both intensity and volume during the second half of the match, suggesting that players experience a drop in physical performance as the game progresses (*p* < 0.05) **A:** Total Distance, **B:** Mean Metabolic Power **C:** Total Energy Expenditure, **D:** High Power Energy Expenditure.

Comparing data from the first and second half of the matches, mean metabolic power (10.36 ± 1.03 W/kg vs. 9.59 ± 0.97 W/kg, d = 1.24, strong effect), total distance (5,260 ± 296 m vs. 5,127 ± 361 m, d = 0.04, no effect), total energy expenditure (28.39 ± 3.30 kJ/kg vs. 27.11 ± 4.29 kJ/kg, d = 0.48, small effect) and high-intensity energy expenditure (10.47 ± 2.23 kJ/kg vs. 9.52 ± 2.35 kJ/kg, d = 0.62, medium effect) were higher in the first half compared to the second for all players and every single position, respectively (*p* < 0.05) (Figure 1).

## Discussion

The aim of this study was to investigate the positional differences in metabolic power and metabolic power-derived parameters, and to compare the traditional speed-based approach with the metabolic power approach in describing the intensity and a volume of match-play. The study revealed two major findings. First (1), position-specific differences in volume and intensity relative parameters do exist. Second (2), classification of high intensity based on speed or acceleration underestimates the time spent and energy expended high intensity. The findings of differences (between positions) in volume and intensity related parameters lead to a verification of hypothesis (1) and higher values for time spent and energy expended at high metabolic power verifies the secondary hypothesis.

Average distance covered (10,531 m) in this study is similar to comparable studies investigating movement and intensity profiles in soccer in other leagues where it ranges from 10,746 to 10,950 m (Osgnach et al., 2010; Di Salvo et al., 2013; Mallo et al., 2015; Altmann et al., 2021). EE (57 kJ kg^−1^), however, was a little lower in the German Bundesliga compared to the Italian Serie A (61 kJ kg^−1^; Di Salvo et al., 2013; Mallo et al., 2015; Osgnach et al., 2010). Run activities in the German Bundesliga seem to be a little less intermittent (EDI: 1.16) compared to the Italian Serie A (EDI: 1.20). Using a fixed metabolic power threshold of 20.0 W kg^−1^ as suggested by (Polglaze et al., 2018) revealed a clearly higher energy expenditure from anaerobic sources in the German Bundesliga (AI: 0.35) compared to the Italian League (AI: 0.18). In order to analyze individual energy demands more precisely, further investigations should determine the individual metabolic power threshold and integrate this individual value into the analysis.

Using equivalent distance index as parameter for intermittency, soccer (EDI: 1.16) seems to be less intermittent compared to rugby [1.28; (Kempton et al., 2015)] and hockey [1.24; (Polglaze et al., 2018)], but more intermittent than Australian football [1.10; (Coutts et al., 2015)]. One explanation for the differences in intermittency between these team sports may be the player density (i.e., area per player, neglecting goalkeeper), which is highest in Australian football (924 m^2^/player), followed by soccer (357 m^2^/player), hockey (251 m^2^/player), and rugby (233 m^2^/player). Generally speaking, the less space a player has on the field, the more erratically he has to move in order to be clear of his opponents. Another explanation might be differences in playing time and the number of substitutions in the game. Official playing time is shortest in hockey (2 min × 35 min), followed by Australian football (4 min × 20 min) and rugby (2 min × 40 min), and soccer (2 min × 45 min), however, net playing time differs, because the game clock does not stop when the ball is out of play in soccer, hockey and rugby, unlike Australian football. The number of substitutions is unlimited in rugby, hockey and Australian football, while it was limited to 3 players until 2019 in soccer. The shorter the playing time for one individual player, the higher the intermittency of his movements might be because the player has enough time for regeneration during his time on the substitute bench. Further, sport-specific movements also explain differences in intermittency.

### Positional differences

Various differences in metabolic power parameters between positions were found, which implies a great variability in the metabolic and running profile and demands between these positions. For CB, for instance, who have 1) the lowest value in each metabolic power parameter and 2) the smallest AI (0.32), a soccer game seems to be a more steady-state based activity. In comparison to the other positions, energy seems to be mostly gathered out of oxidation processes. As indicated by a higher EDI, the movements of WM seem to be more intermittent and erratic. However, there is no significant difference in the absolute energy expenditure between CB, ST, WM and WB. Thus, from an energetic perspective and if playing intensity is defined as total energy expenditure per time, playing intensity is similar between positions. Central attacking midfielders and central midfielders cover the most distance followed by WM, WB, ST and CB. This sequence contrasts studies of top-class soccer players from the Spanish, Italian and English leagues, where WM cover the greatest distance (Di Salvo et al., 2007; Rampinini et al., 2007; Bradley et al., 2010; Dellal et al., 2010; Mallo et al., 2015). Over the last decade technical and tactical factors evolved more than physiological attributes (Barnes et al., 2014), and except for CB the other playing positions change frequently their positions throughout a soccer match. This might explain similarities in the energetic and movement profiles of the players of these positions (Hoppe et al., 2017).

### High intensity classification

The distance covered at high speed, e.g., while running faster than 15.5 km/h, did not differ between the midfield positions (CAM, CM and WM), but was higher in the midfield compared to the other positions. These findings are in line with previous studies (Di Salvo et al., 2007; Rampinini et al., 2007; Bradley et al., 2010; Dellal et al., 2010; Mallo et al., 2015). The three midfield positions also covered more distance, spent more time and expended more energy over the high metabolic power threshold. Both kinds of analysis indicate that position-group-specific training programs for the development of individual player’s athletic performance should be applied in order to prepare the players the best possible for the demands of each single position. Non-etheless, using the novel metabolic power approach for the analysis of parameters of high intensity energy demands revealed position-specific differences that would not be detected with the traditional speed-category analysis alone. Time spent, distance covered and energy expended over the high-intensity thresholds is higher in every position when metabolic power is used to characterize high-intensity movements (48.9% more time; 66.5% more distance, 50.4% more energy). This is in line with international hockey matches (Polglaze et al., 2018) and soccer training drills (Gaudino et al., 2013).

In comparison of high-intensity activities for speed and metabolic power every activity over 15.5 km/h is classified as high intense for the former whether the athlete accelerates or not. Therefore, running under 15.5 km/h is not considered as high-intense from the traditional speed-based perspective, even if the acceleration is very high. Most high-intense metabolic power activities are short little bouts where the acceleration is high while starting at a different speed, the higher the starting speed the lower the needed acceleration to exceed 20 W/kg (Osgnach et al., 2010). A small deceleration while running at a high speed leads to a large reduction in energy cost (Polglaze et al., 2018). As seen from the data the acceleration (above the fixed threshold used in this study) cannot directly be compared to metabolic power because it only accounts for a small proportion of high-intensity energy expenditure (Sonderegger et al., 2016; Polglaze et al., 2018).

Maximal accelerations are the most energetically demanding elements in team sports, however, the majority of them are excluded when intensity is characterized according to speed. When using this approach, the effects of accelerations are significantly reduced because 34% of sprint efforts are preceded by a maximal acceleration, while when starting an accelerated sprint, the speed is still low (Varley and Aughey, 2013). Thus, when categorizing high-intensity activities only by speed, the high intensity demands are clearly underestimated. Therefore, inclusion of metabolic power in match analysis is urgently necessary.

### Difference of halftimes

Waldron and Highton (2014) reported that distance and speed decrease in the second half of a match. This, however, is not necessarily a clear indicator of a reduction of playing intensity, because, as mentioned above, intermittent loads are not reflected in this speed-based analysis. However, as our analysis of metabolic power also revealed a decline of total energy expenditure, energy expenditure over the metabolic power threshold, and mean metabolic power in the second halves of the matches, fatigue clearly seems to occur in the second halves and towards the end of the matches (Mohr et al., 2003). The reduction of metabolic and running performance could also lead to a reduction in skill-based activities like less shots or successful passes (Rampinini et al., 2009). It would be interesting to know if players with a high individual aerobic-anaerobic threshold experience less fatigue in the second halves of the matches.

### Limitations

The focus in this study was the evaluation of positional differences. In soccer, players change their positions frequently depending on the tactical strategy of the match, however, this study did not account for variations in the tactical behavior nor difference tactical formations (e.g., 4-3-3; 4-5-1; 3-5-2). Further, the metabolic power approach takes into account horizontal movements of the center of mass of the players’ body, while any vertical movements like jumping or movements of the limbs while passing the ball or shooting, which cause additional metabolic investments, are neglected. Actions like shots, passes and tackles yield a certain amount of energy and impact the overall energy expenditure. These actions need further evaluation and are not implemented in the metabolic power approach yet. Additionally, the polynomial equation for the analysis of metabolic power needs to be evaluated group-specifically (Buglione and di Prampero, 2013; di Prampero and Osgnach, 2018; Savoia et al., 2020). Further, this study analyzed the data from three teams of the German Bundesliga and their respective opponents. Therefore, the sample size for bottom- and top-ranked teams was different, which reduced the power in the statistical analyses. Also, the run- and energetic profiles of the bottom-ranked could differ when facing similar less-successful opponents compared to the analyses of matches against top- or mid-ranked teams.

### Practical relevance

The differences in intensity and volume parameters derived from metabolic power during soccer match-play indicates the importance of tailoring training to each player’s positional profile. Incorporation metabolic power analysis in soccer is beneficial and imperative to optimize daily training work and for the advancement and development of the sport. Metabolic power helps in monitoring training load especially when individual thresholds are included in the analysis. In soccer particularly, high-intensity efforts are characterized by brief bursts, where time and distance may not accurately capture these activities and the turnover rate of adenosine triphosphate can be extremely high (Polglaze and Hoppe, 2019).

### Future directions

The presents study summarized volume and intensity parameters derived from the metabolic power approach and showed differences among positions. However, the metabolic power model has limitations in accurately depicting physical and physiological demands. Future research should focus on implementing soccer specific actions like passing and shooting in the description of external load. Also, analysis of a more player-specific instead of position-specific approach could be the topic of future research.

## Conclusion

This study confirms that positional differences exist for both metabolic power- and running-based parameters. Mainly, central midfield positions showed higher volume and intensity in soccer match-play and wide midfielders play is more intermittent in nature. This indicates the need for position-specific athletic training regimes. The traditional speed-based approach to characterize intensity showed lower values in time spent, distance covered and energy expended in high-intensity domains compared to the metabolic power approach, suggesting an underestimation of the respective parameters. Metabolic power incorporates both speed and acceleration and might be more appropriate to describe the demands of intermittent and variable-run characteristics of team sports. As the external validity of the metabolic power approach regarding energy expenditure might still be in a question, it acts as a useful monitoring tool in team sports and might be more accurate for describing volume and intensity than the traditional approach. Nevertheless, the metabolic power approach requires further adjustments when it comes to the individualization of training measures and to the determination of overall external soccer match load as it only considers locomotion and no further soccer specific forms of movements beside running.

## Data Availability

The data analyzed in this study is subject to the following licenses/restrictions: Restrictions apply to the availability of these data, as they are property of the Deutsche Fußball Liga (DFL) and only accessible for the participating members. Requests to access these datasets should be directed to ext.hendrik.weber@dfl.de.
